# RVFNet: 4D mmWave Radar-Vision Fusion Multi-Task Panoptic Perception Network for Unmanned Surface Vehicles

**DOI:** 10.3390/s26216953

**Published:** 2026-11-03

**Authors:** Fangxiang Tang, Bing Tang, Shenbo Liu, Dongxue Zhao, Lijun Tang, Junlong Tang

**Affiliations:** 1 School of Physics and Electronic Science, Changsha University of Science and Technology, Changsha 410114, China; 24111032126@stu.csust.edu.cn (F.T.); 24111022103@csust.edu.cn (B.T.); liusb@csust.edu.cn (S.L.); zhao-dx@stu.csust.edu.cn (D.Z.); 2 Hunan Province Higher Education Key Laboratory of Modeling and Monitoring on the Near-Earth Electromagnetic Environment, Changsha 410114, China; 3 Hunan Provincial Key Laboratory of Flexible Electronic Materials Genome Engineering, Changsha University of Science and Technology, Changsha 410114, China

**Keywords:** 4D mmWave radar, radar-vision fusion, multi-task perception, Unmanned Surface Vehicles

## Abstract

4D mmWave radar-vision fusion is a low-cost and promising solution for Unmanned Surface Vehicles navigation. However, existing methods mainly focus on single tasks and suffer from insufficient radar feature extraction and inefficient multi-modal fusion. This paper proposes RVFNet, a 4D mmWave radar-visual fusion multi-task perception network. It designs the Radar Feature Enhancement Module (RFEM) and alternately stacks RFEM with conventional convolution blocks to construct the Radar Feature Extraction Network (RFENet), which solves the problem of insufficient extraction of sparse and irregular radar point cloud features and suppresses water-surface clutter; it constructs the Multi-modal Feature Adaptive Fusion Module (MFAFM) with content-guided attention. MFAFM generates dynamic modal weights and adaptively balances visual and radar information to alleviate missed detection and false detection in harsh scenarios. Compared with mainstream methods, experimental results on the WaterScenes dataset show that RVFNet requires only 3.3 GFLOPs while achieving an mAP_50_ of 79.0%, an mAP_50–95_ of 46.7%, an mIoU of 84.0% for object segmentation, and an mIoU of 98.8% for drivable-area segmentation, demonstrating superior overall perception performance. Cross-scenario experiments on the FloW-RI water-surface dataset and the nuScenes v1.0-trainval autonomous-driving dataset further verify the strong generalization capability of RVFNet, demonstrating its potential for deployment on embedded devices.

## 1. Introduction

Unmanned Surface Vehicles (USVs) are important tools for conducting tasks such as wide-area water-surface surveillance, emergency rescue, environmental monitoring, and garbage removal. To identify various targets such as surface ships, buoys, bridge piers, people overboard, and floating garbage, it is necessary to develop a high-precision and low-latency panoptic perception system [1]. RGB cameras can provide rich color information, fine-grained textures, and target contours, offering strong semantic representation capabilities. However, they have the following problems: (1) surface visible light images are prone to degradation due to external factors such as rain, fog, motion blur, and changes in lighting, resulting in reduced image contrast and loss of feature information; (2) the proportion of small targets on the water-surface is high, and there is less effective feature information, making it difficult to judge visual textures; (3) background noise caused by waves, reflections, and wake flows can interfere with the objects of interest and affect the stability of detection [2]. These various factors pose a series of challenges to camera sensors, making it difficult for visual sensors to achieve high-precision panoptic environmental perception.

4D mmWave radar has advantages such as long wavelengths [3] and long detection ranges [4]. It can penetrate and propagate through rain, fog, snow and other adverse weather conditions with low attenuation [5,6,7], and is less affected by harsh lighting conditions such as strong light and darkness [3]. However, the radar point cloud has the characteristics of sparse features and irregular distribution, and the radar sensor has a low resolution, which can only obtain the spatial 4D information of the target, such as range, velocity, azimuth angle, and height, lacking the texture and color information of the target, and it is difficult to achieve precise target recognition [8,9]. Therefore, integrating 4D mmWave radar with visual sensors can improve the accuracy and robustness of all-weather autonomous perception for USVs [10,11].

In dynamic and complex water-surface environments, RGB images and radar signals differ significantly in their information representation and reliability. Simple concatenation is unable to dynamically adjust the information contribution of the two modalities according to different weather, lighting conditions and target states. Therefore, for the heterogeneous modality information of water-surface scenes, designing a cross-modal adaptive fusion collaboration mechanism is of great significance for improving the perception ability of USVs in complex environments [12].

In recent years, radar-vision fusion has mainly been applied to single-task perception, where multi-modal information is fused to improve the performance of single tasks, such as object detection or semantic segmentation. However, a single task ignores the rich correlation information contained in tasks and has weak generalization ability, while multi-tasks share representations and parameters, and through information interaction, it is beneficial to enhance the all-weather environmental perception ability of USVs [13].

This paper explores methods for extracting irregular and sparse features from radar point cloud information, studies the mechanism of heterogeneous information fusion between radar and vision, constructs a multi-modal multi-task collaborative perception framework for radar and vision, and designs a multi-modal multi-task water-surface detection network RVFNet. These efforts aim to provide certain technical support for the realization of USVs with high-precision and high-robustness perception systems. The contributions of this paper are as follows:

1. Considering the sparse and irregular distribution characteristics of 4D mmWave radar point clouds, this paper designs the Radar Feature Enhancement Module (RFEM) and alternately stacks RFEM with conventional convolution blocks to construct the Radar Feature Extraction Network (RFENet). RFEM collaboratively extracts the features of irregular target areas through standard convolution and deformable convolution, and combines Channel Shuffle and Efficient Channel Attention (ECA) to interact and filter multi-dimensional radar features, focusing on the effective target echo features and suppressing the interference of water-surface clutter, in order to enhance the representational ability of the radar feature map.
2. To address the issues of inadequate fusion of radar and RGB features and the unbalanced contribution of dual-modal information in different scenarios, the Multi-modal Feature Adaptive Fusion Module (MFAFM) is proposed. The module generates dynamic modal weights through Content-guided attention jointly constructed by channel, spatial and pixel three-level attention, realizes the dynamic balance and complementary fusion of dual-modal information, and solves the problem of missed detection and false detection in harsh scenes.
3. Comprehensive comparative experiments were conducted on the dedicated multi-modal datasets for water-surface perception, WaterScenes and FloW-RI, as well as the autonomous driving dataset nuScenes v1.0-trainval. The results show that, under the unified experimental configuration, RVFNet has significant advantages over the existing mainstream advanced algorithms. It achieves excellent results in the drivable area segmentation task and achieves the best performance in the object detection task and object semantic segmentation task.

The main content arrangement of this article is as follows: Section 2 introduces the related work; Section 3 presents the proposed RVFNet model in this paper; Section 4 presents extensive experiments and analysis and analyses; Section 5 summarizes the entire paper and looks forward to future work.

## 2. Related Work

### 2.1. Radar Feature Extraction

The existing radar-RGB fusion models usually employ structurally symmetrical and relatively independent encoders to extract features of different modalities, which can effectively avoid the interference of heterogeneous data when directly mixed; in recent years, several approaches have been proposed for radar feature extraction. Sun et al. proposed CaRaFFusion, which uses PointNet as the radar encoder to directly process the raw radar point cloud and map the physical attributes such as coordinates and velocity to obtain point embedding features [14]. This solution does not require projecting the sparse radar point cloud into a two-dimensional grid, fully preserving the original point-level information, and can jointly represent multiple physical quantities such as spatial position, velocity, and scattering characteristics. However, PointNet only performs feature transformation independently for a single point and lacks a neighborhood aggregation mechanism, which makes it unable to model the local spatial context relationship between radar points. Another method is to project the radar point cloud into a pseudo-image and directly use a general CNN encoder. However, the fixed sampling pattern of conventional convolution is difficult to adapt to the sparse and irregular spatial distribution characteristics of 4D mmWave radar point clouds, easily resulting in insufficient representation of effective radar points and their local geometric structures. In response to this problem, Yin et al. proposed WS-DETR, which uses SMPConv to construct an independent radar encoder based on REVP pseudo-images and employs learnable sampling positions to adapt to variations in point cloud density [15]. Guan et al. proposed Achelous, which employs average pooling and deformable convolution to address the spatial irregularity of radar point clouds [16]. Both WS-DETR and Achelous adopt convolutional radar encoders, focusing only on the geometric adaptation of the radar spatial dimension, lacking the joint exploration of multi-dimensional physical channels, and the encoders cannot complete effective feature selection. Guan et al. proposed the Mask-VRDet water-surface panoptic perception model, which employs DBSCAN clustering for external clutter filtering at the input stage and then projects the filtered points into graph nodes for feature aggregation using VR-GCN [17]; Guan et al. proposed ASY-VRNet, which converting RGB images and radar pseudo-images into irregular point sets, and extracting local and contextual radar features through the VRCoC context clustering module [18]. Mask-VRDet and ASY-VRNet introduce clustering and graph structure to model the radar spatial relationships, thereby enhancing the structural expression ability of radar features. However, these methods rely on complex graph construction and clustering operations, which incur substantial computational overhead and limit their applicability to real-time scenarios. Therefore, geometric adaptive convolution operators show considerable potential for radar feature extraction. Motivated by these observations, this paper adopts geometric adaptive convolution operators as the basis of the radar feature extraction network and further introduces multi-dimensional physical channel interaction and internal clutter suppression mechanisms to improve the overall representation quality of radar features.

### 2.2. Multi-Modal Feature Fusion

With the continuous improvement of the complementary perception capabilities of radar and vision, how to effectively achieve the fusion of heterogeneous modal features has become the key issue in multi-modal perception research. Multi-modal fusion can be divided into early fusion, mid-term fusion, late fusion and mixed fusion. In order to make the best use of the underlying correlations between modalities as early as possible and reduce information loss, early fusion strategies are commonly used [19] for multi-modal fusion. WMF-YOLOv7 [20], Achelous, and Achelous++ [21] fuse radar and image information by selecting effective features through attention mechanisms and then concatenating them. However, the fusion process is relatively fixed and has limited complementary capabilities between modalities. To better adapt to the complex and variable water-surface environments, adaptive fusion has become the mainstream solution. PhysFusion [22] and CalibFusion [23] both adopt token-based cross-modal interaction, establishing dynamic correlations between radar and visual features through cross-attention to achieve adaptive multi-modal information fusion. Among them, PhysFusion combines object queries and GRU for multi-frame temporal aggregation, and CalibFusion achieves geometric alignment of radar and image through extrinsic parameter correction and differentiable projection scattering. Cross-attention can utilize the context information of one modality to guide the enhancement of the features of another modality, but its performance depends on the quality of the input features. When there is clutter, noise or insufficient effective information in the radar, it is prone to introduce invalid or interfering information into the cross-modal interaction; at the same time, large-scale token association will also bring high computational overhead. Guan et al. proposed ASY-VRNet with an Asymmetric Fair Fusion (AFF) strategy, using Image-Radar Concatenation (IRC) and Radar-Image Multiplication (RIM) respectively for cross-modal enhancement for detection and segmentation tasks [18]. However, it does not consider the dynamic differences of different modal information in water-surface scenes caused by environmental changes, and is difficult to adaptively adjust the contribution of the two modalities according to the reliability of radar and RGB features in different scenarios. To address the above limitations, this paper proposes an adaptive fusion module that dynamically adjusts the weights of radar and RGB features according to changes in water-surface conditions, thereby achieving effective fusion of heterogeneous features.

## 3. Methodology

### 3.1. Overall Architecture

**Figure 1:**
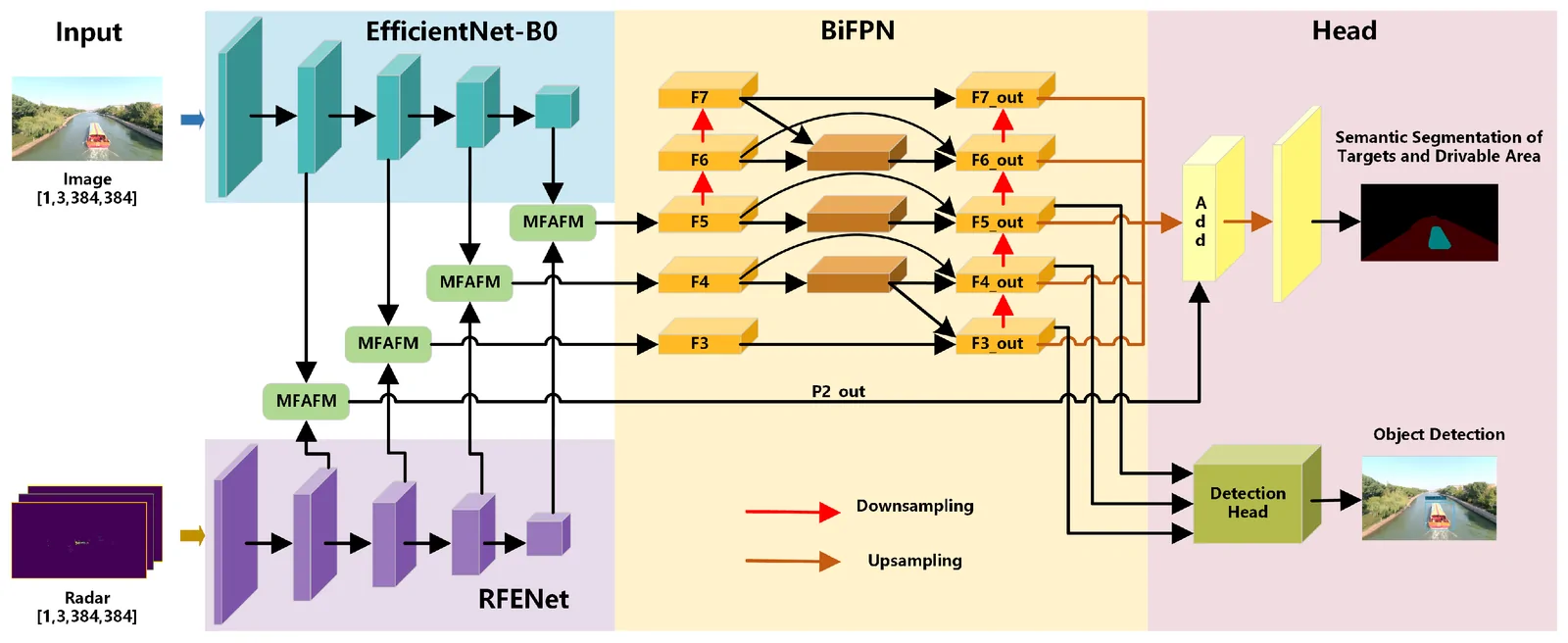
RVFNet architecture.

Figure 1 shows the overall architecture of RVFNet proposed in this paper. This framework adopts a dual-branch parallel encoding structure, which can simultaneously process two types of heterogeneous sensing data: RGB visible light images and 4D mmWave radar point clouds. To achieve multi-modal alignment, the original three-dimensional radar point cloud is projected onto the image plane through coordinate transformation to generate a radar pseudo-image as the input for the radar branch [24]. The overall network architecture is divided into four major parts: dual-path feature encoder, cross-modal fusion network, neck network, and dual-task dedicated decoder. These components work together under a unified network to jointly optimize the two perception tasks of object detection and pixel-level semantic segmentation. The semantic segmentation task is accomplished through a unified 9-class segmentation output, simultaneously predicting multiple types of water surface targets and drivable areas.

In the feature extraction stage, the RGB images are processed by the lightweight image encoder EfficientNet-B0 [25], while the transformed radar pseudo-images are handled by the self-designed Radar Feature Extraction Network (RFENet). Eventually, the heterogeneous features processed by the dual encoders are obtained. These heterogeneous features are input into the radar-visual fusion network, which contains the Multi-modal Feature Adaptive Fusion Module (MFAFM) for dynamically adjusting complementary information. This fusion representation forms the shared feature space of RVFNet, which serves as the shared feature representation for downstream multi-task prediction.

The neck network adopts the Bidirectional Feature Pyramid Network (BiFPN) [26]. The fused features from the backbone network are fed into the neck network. The traditional unidirectional Feature Pyramid Network (FPN) only transmits features from top to bottom in a single direction, and there are limitations in the cross-scale information flow; while BiFPN builds a bidirectional path from top to bottom and bottom to top, it adds learnable weights to different resolution features, adaptively balancing the contribution degree of features of different scales, significantly enhancing the multi-scale representation ability of small and distant targets, and this structure is adopted as the neck network to meet the requirements of water-surface perception.

During the task decoding stage, the multi-scale fused features output by BiFPN are respectively sent to the detection head and the segmentation head: the detection head outputs the target bounding box, category and confidence, completing the location and identification of water-surface targets; the segmentation head outputs the pixel-level mask.

### 3.2. Input Data

The original input data consists of two modalities: the RGB images $I\in R^{3\times H\times W}$ captured by the monocular camera, and the three consecutive frames of radar point clouds $P\in R^{N\times 6}$ generated by the 4D mmWave radar in the radar coordinate system. The radar point clouds contain six physical quantities $\left(X_{r},Y_{r},Z_{r},r,v,p\right)$, representing the three-dimensional spatial coordinates, range, velocity and power respectively. To facilitate the fusion operation of the radar point clouds and the RGB images, the radar point clouds are transformed from the radar coordinate system to the camera imaging plane. This coordinate transformation is achieved based on the extrinsic parameter matrix E between the two types of sensors and the camera intrinsic parameter matrix K, as shown in Equations (1) and (2) [27].

(1) $${\left[X_{c},Y_{c},Z_{c},1\right]}^{T}=E\cdot {\left[X_{r},Y_{r},Z_{r},1\right]}^{T}$$

(2) $${\left[u,v,1\right]}^{T}=\frac{1}{Z_{c}}\cdot K\cdot {\left[X_{c},Y_{c},Z_{c}\right]}^{T}$$

In Equation (1), ${\left[X_{c},Y_{c},Z_{c},1\right]}^{T}$ represents the homogeneous coordinates of the point in the camera coordinate system, and ${\left[X_{r},Y_{r},Z_{r},1\right]}^{T}$ represents the homogeneous coordinates of the point in the mmWave radar coordinate system. In Equation (2), ${\left[u,v,1\right]}^{T}$ represents the homogeneous coordinate of the point in the image coordinate system.

After obtaining the radar projection points (u,v) in the camera plane from Equation (2), the 2D radar point cloud features are concatenated along the channel dimension to generate a radar pseudo-image. This radar pseudo-image contains three feature channels: range, velocity, and power. In this paper, the time stamp is used to complete the time synchronization between the camera image and the multi-frame radar point cloud. Three consecutive frames of radar point cloud near the image acquisition time are selected, and the self-motion compensation is completed by combining the position and orientation information of the unmanned surface vehicle. In terms of sensor calibration, this paper uses the calibration external provided by the data set to participate in the camera internal parameters. The calibration error of the external parameters is determined by the original calibration process of the data set, and no additional correction is required in this paper. When generating radar pseudo image by projection, if multiple radar points are projected to the same pixel position, the measurement value from the closest point is taken as the pixel feature. For empty pixels without radar point projections, zeros are directly filled. The radar projection point (u,v) serves as the spatial index on the radar pseudo-image, while the three feature channels store the original physical measurement values of the radar, namely range, velocity, and power.

### 3.3. Radar Pseudo-Image Encoder

**Figure 2:**
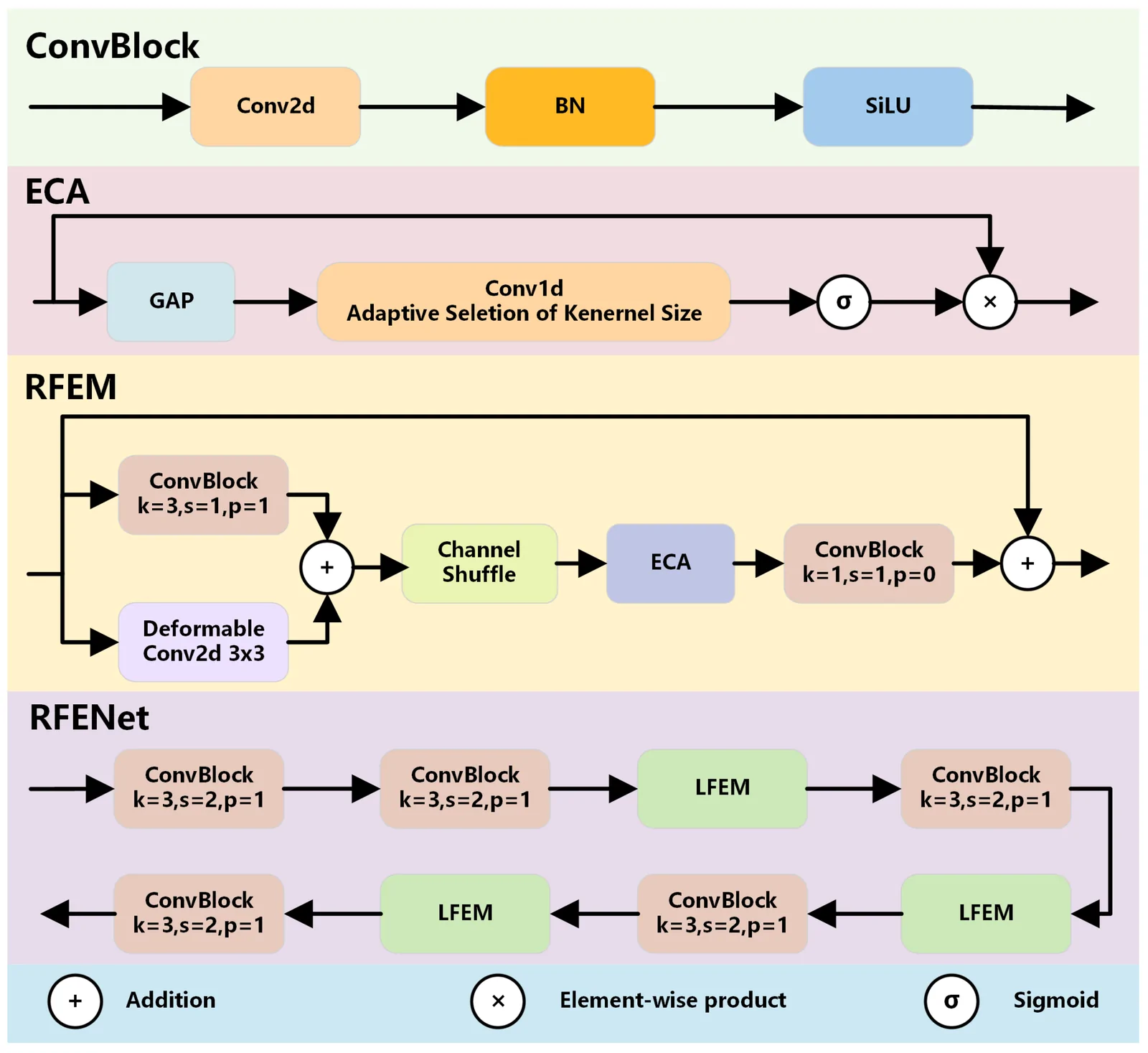
Architecture of RFENet and its constituent modules.

The radar pseudo-image encoder is responsible for extracting features from the radar point cloud. The radar point cloud has the characteristics of being sparse and irregularly distributed. After projecting the three-dimensional radar point cloud onto a two-dimensional image plane, the radar pseudo-image contains a large number of blank areas of numerical values, and only the positions of the target and clutter reflection points contain the point cloud features. Therefore, the Radar Feature Enhancement Module (RFEM) is designed as the core unit of the Radar Feature Extraction Network (RFENet). The overall processing flow of RFEM is divided into four steps, and the structure is shown in Figure 2.

Step 1: Parallel dual-branch feature extraction is implemented. The radar pseudo-image $f_{r}\in R^{C\times H\times W}$ is input into two parallel extraction branches: one uses a standard 3×3 convolution to effectively extract the local texture information and spatial structure features in $f_{r}$ through a fixed receptive field; the other uses 3×3 deformable convolution [28], which dynamically learns the sampling offset position to enable the convolution kernel to adaptively match the irregular spatial morphology of the radar point cloud. The two branches respectively obtain two features, $f_{\mathrm{conv}3}$ and $f_{\mathrm{dconv}}$, as shown in Equations (3) and (4).

(3) $$\begin{array}{c} f_{\mathrm{conv}3}=\mathrm{ReLU}\left(\mathrm{BN}\left({\mathrm{Conv}}_{3\times 3}\left(f_{r}\right)\right)\right),f_{\mathrm{conv}3}\in R^{C_{0}\times H\times W} \end{array}$$

(4) $$\begin{array}{c} f_{\mathrm{dconv}}=\sum_{k=1}^{K}w_{k}\cdot f_{r}\left(p+p_{k}+\Delta p_{k}\right)\cdot \Delta m_{k},f_{\mathrm{dconv}}\in R^{C_{0}\times H\times W} \end{array}$$

In Equation (4), K=9, indicating the number of deformable convolution sampling points. *p*, $p_{k}$ and $\Delta p_{k}$ represent the positions of the center point of the convolution kernel on the input feature map $f_{r}$, the predefined offset at position *k* and the learnable offset at position *k*, where $w_{k}$ and $\Delta m_{k}$ are the learnable weights and modulation coefficients at position *k*.

Subsequently, the features from the two branches are fused element-wise to fully combine the local texture extraction capability of the standard convolution and the adaptability of the deformable convolution to irregular target structures, thereby obtaining radar features with stronger discriminative ability. The calculation formula is as shown in Equation (5).

(5) $$f_{\mathrm{out}}=f_{\mathrm{conv}3}+f_{\mathrm{dconv}},f_{\mathrm{out}}\in R^{C_{0}\times H\times W}$$

Step 2: Channel Shuffle promotes cross-dimensional information interaction [29]. The fused radar features are subjected to the Channel Shuffle operation. The radar pseudo-image stores three independent physical information types (range, velocity, and power) separately. The combined features still consist of three independent physical information channels, and the features from different physical dimensions lack information flow. By reorganizing and reassigning the channel order with Channel Shuffle, the original channel arrangement is disrupted, allowing the radar range, velocity, and reflection intensity information to flow among each other, breaking the isolation of different physical features and fully integrating multi-dimensional radar information, as shown in Equation (6).

(6) $$f_{\mathrm{shuffle}}=\mathrm{ChannelShuffle}\left(f_{\mathrm{out}}\right),f_{\mathrm{shuffle}}\in R^{C_{0}\times H\times W}$$

Step 3: Channel screening via ECA [30]. After Channel Shuffle, the features $f_{\mathrm{shuffle}}$ are filtered using ECA to obtain the effective radar features. Firstly, the feature map undergoes Global Average Pooling (GAP) to compress the spatial dimensions, resulting in a one-dimensional channel description vector, as shown in Equation (7). Then, through adaptive one-dimensional convolution, the adjacent channel dependencies are captured, and the weights for each channel are generated using Sigmoid. Element-wise weighting is applied to correct the original features, automatically suppressing the cluttered ineffective channels and strengthening the target-related physical channels, as shown in Equations (8) and (9). Subsequently, 1×1 convolution is used to achieve cross-channel information fusion, introducing nonlinear transformation while maintaining the spatial dimensions of the feature map, further enhancing the radar features after attention filtering, as shown in Equation (10).

(7) $$\begin{array}{c} z=\mathrm{GAP}\left(f_{\mathrm{shuffle}}\right),z\in R^{C_{0}\times 1\times 1} \end{array}$$

(8) $$\begin{array}{c} w_{\mathrm{eca}}=\sigma \left(\mathrm{Conv}1D_{k}\left(z\right)\right),w_{\mathrm{eca}}\in R^{C_{0}\times 1\times 1} \end{array}$$

(9) $$\begin{array}{c} f_{\mathrm{eca}}=w_{\mathrm{eca}}⊙f_{\mathrm{shuffle}},f_{\mathrm{eca}}\in R^{C_{0}\times H\times W} \end{array}$$

(10) $$\begin{array}{c} f_{\mathrm{conv}1}=\mathrm{ReLU}\left(\mathrm{BN}\left({\mathrm{Conv}}_{1\times 1}\left(f_{\mathrm{eca}}\right)\right)\right),f_{\mathrm{conv}1}\in R^{C\times H\times W} \end{array}$$

Here, σ stands for the Sigmoid activation function, and ⊙ denotes element-wise multiplication operation.

Step 4: Residual connection retains feature details. ECA output features, after 1×1 convolution, the output $f_{\mathrm{conv}1}$ and the original input of RFEM are added to construct the residual structure to prevent the loss of shallow radar feature details. Convolutions are added with the original input of RFEM to construct a residual structure to prevent the loss of shallow radar feature details. Finally, the output is $f_{\mathrm{rfem}}$, as shown in Equation (11).

(11) $$f_{\mathrm{rfem}}=f_{\mathrm{conv}1}+f_{r},f_{\mathrm{rfem}}\in R^{C\times H\times W}$$

RFENet is composed of multiple layers of $f_{\mathrm{conv}3}$ and RFEM alternately stacked together, extracting irregular radar features layer by layer.

### 3.4. Radar-Visual Fusion Network

**Figure 3:**
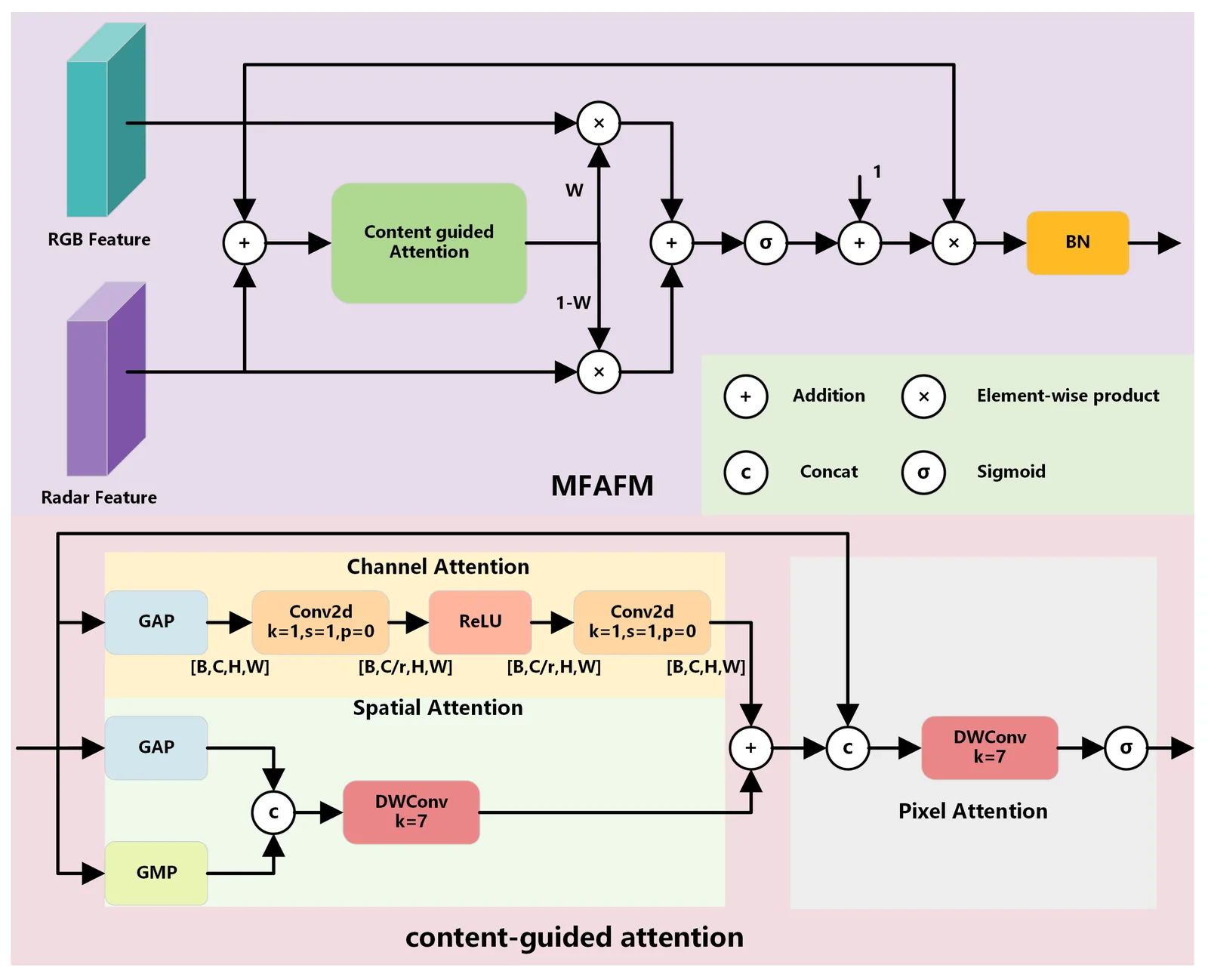
The Multi-modal Feature Adaptive Fusion Module.

In the water-surface scene, the radar point cloud information is sparse and irregular, and the visible light image is prone to texture degradation due to strong reflection from the water-surface and water mist interference. Therefore, this paper designs the Multi-modal Feature Adaptive Fusion Module (MFAFM). The input of MFAFM is the last four feature maps output by the image encoder and the radar encoder. The output of MFAFM is four adaptively balanced multi-modal fusion features, which integrate high-level features of visual semantics and radar physical representations and are collectively referred to as sensor features. The overall structure is shown in Figure 3. The complete processing flow of MFAFM is divided into three stages: content-guided attention, adaptive weighted fusion, and gated residual.

Given the image feature $f_{v}\in R^{C\times H\times W}$ and the radar feature $f_{r}\in R^{C\times H\times W}$, first, add the two sets of features element-wise to obtain the multi-modal fusion feature $f_{\mathrm{vr}}$, as shown in Equation (12). $f_{\mathrm{vr}}$ is fed into the content-guided attention mechanism, and dynamically learned modality complementary weights are shown in Equations (13)–(17). Content-guided attention utilizes two parallel attention branches (one for channels and one for space) and then sequentially applies the pixel-level attention to fully exploit the global channel dependencies, spatial positional correlations, and fine-grained pixel-level contour information. After fusion, the scene dynamic weights $W\in R^{1\times H\times W}$ are generated.

(12) $$\begin{array}{c} f_{\mathrm{vr}}=f_{v}+f_{r},f_{\mathrm{vr}}\in R^{C\times H\times W} \end{array}$$

(13) $$\begin{array}{c} \mathrm{ChannelAtt}\left(\cdot \right)={\mathrm{Conv}}_{1\times 1}(\mathrm{ReLU}\left({\mathrm{Conv}}_{1\times 1}\left(\mathrm{GAP}(\cdot )\right)\right)) \end{array}$$

(14) $$\begin{array}{c} \mathrm{SpatialAtt}\left(\cdot \right)={\mathrm{DWConv}}_{7\times 7}(\mathrm{Concat}\left(\mathrm{GAP}(\cdot )+\mathrm{GMP}(\cdot )\right)) \end{array}$$

(15) $$\begin{array}{c} \mathrm{PixelAtt}\left(\cdot \right)={\mathrm{DWConv}}_{7\times 7}\left(\cdot \right) \end{array}$$

(16) $$\begin{array}{c} f_{Att}=\mathrm{PixelAtt}(\mathrm{Concat}(\left(\mathrm{ChannelAtt}\left(f_{\mathrm{vr}}\right)+\mathrm{SpatialAtt}\left(f_{\mathrm{vr}}\right)\right),\;f_{\mathrm{vr}})) \end{array}$$

(17) $$\begin{array}{c} W=\mathrm{Sigmoid}\left(f_{Att}\right),W\in R^{1\times H\times W} \end{array}$$

Then, the adaptive weighted fusion stage is entered. Based on the dynamic weight W, the dual-modal features are adaptively scaled and corrected. The correction formulas are as in Equations (18) and (19):

(18) $$\begin{array}{c} {\hat{f}}_{v}=f_{v}⊙W,{\hat{f}}_{v}\in R^{C\times H\times W} \end{array}$$

(19) $$\begin{array}{c} {\hat{f}}_{r}=f_{r}⊙\left(1-W\right),{\hat{f}}_{r}\in R^{C\times H\times W} \end{array}$$

In the formula, ⊙ represents element-wise multiplication operation. The two types of weighted corrected features are added element-wise to obtain the preliminary fused feature $f_{\mathrm{fusion}}\in R^{C\times H\times W}$, as shown in Equation (20).

(20) $$f_{\mathrm{fusion}}={\hat{f}}_{v}+{\hat{f}}_{r},f_{\mathrm{fusion}}\in R^{C\times H\times W}$$

Finally, the gated residual operation is carried out. Since the features of 4D mmWave radar mainly consist of sparse geometric information such as speed, they only have the ability to locate the target and do not possess pixel-level texture and color information. They are difficult to effectively supplement the surface details. Therefore, the original visual features are introduced as the residual branch to supplement the fine-grained texture details. At the same time, the Sigmoid function is used to constrain the feature value range, which can dynamically determine how much of the original information to retain and how much new information to filter. The Batch Normalization (BN) layer stabilizes the distribution of multi-modal fusion features and eliminates the numerical offset and distribution oscillation caused by multi-modal superposition. Finally, the fused features are output, and the calculation is shown in Equation (21).

(21) $$f_{s}=\mathrm{BN}(f_{v}⊙\left(\mathrm{Sigmoid}(f_{\mathrm{fusion}})+1\right)),f_{s}\in R^{C\times H\times W}$$

### 3.5. Detection Head

**Figure 4:**
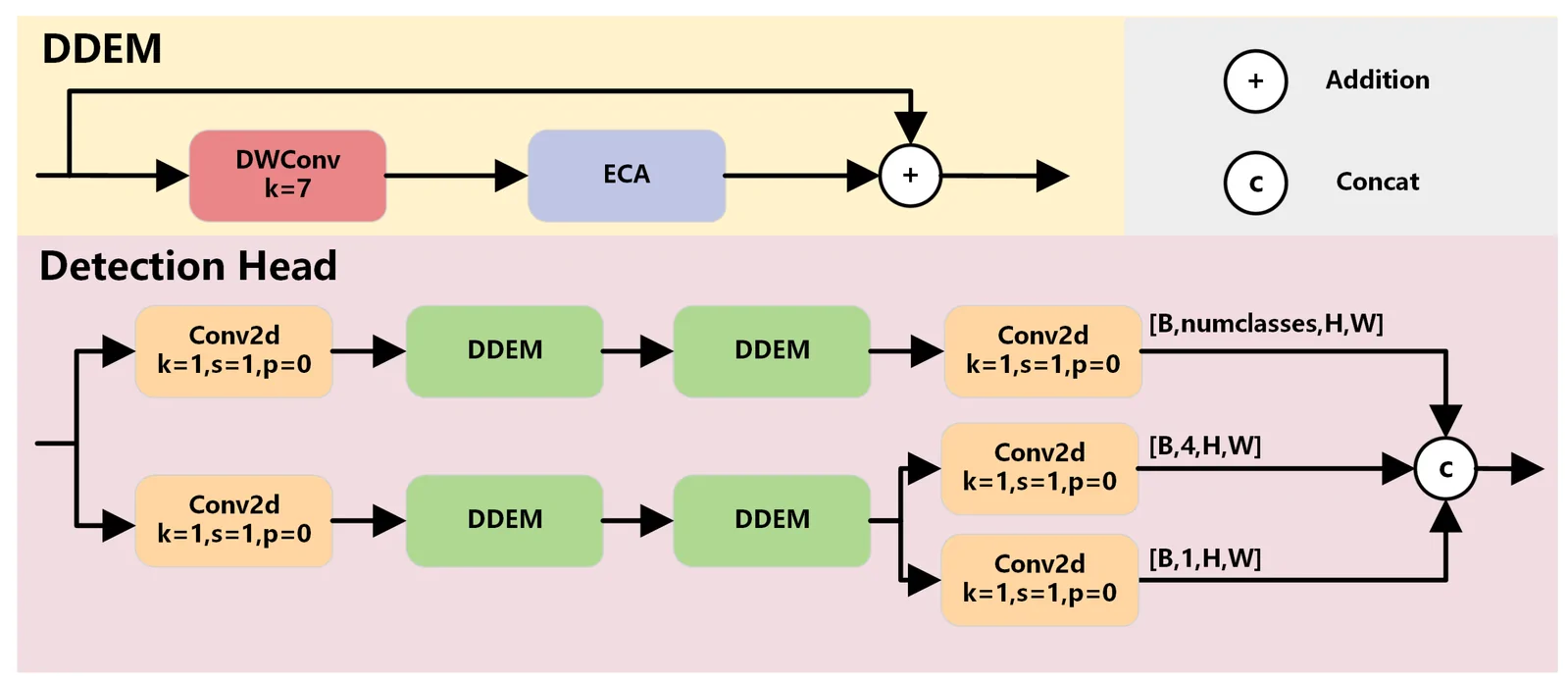
Architecture of the detection head and its DDEM module.

The object detection head adopts a decoupled head design, separating the classification task from the regression task. The classification branch outputs category predictions, while the regression branch outputs bounding box predictions and confidence predictions, as shown in Figure 4. This paper takes the three-layer multi-scale features $\left\{P_{3\_\mathrm{out}},P_{4\_\mathrm{out}},P_{5\_\mathrm{out}}\right\}$ output by the BiFPN neck network as the input of the object detection head. The Depthwise-ECA Detection Enhancement Module (DDEM) is the core part of the object detection head. The input features $f_{\mathrm{input}}\in R^{C_{\mathrm{in}}\times H_{x\_\mathrm{out}}\times W_{x\_\mathrm{out}}},\;x\in \left\{3,4,5\right\}$ first undergo 7×7 depth convolution to capture the large-scale local spatial context; then they are input into ECA to suppress invalid features and enhance target-related features; finally, they are fused with the original features through residual connections to alleviate the problem of gradient attenuation. This module jointly optimizes features from the spatial and channel dimensions to improve the model’s ability to recognize and locate water-surface targets, as shown in Equation (22).

(22) $$f_{\mathrm{DDEM}}\left(f_{\mathrm{input}}\right)=f_{\mathrm{input}}+\mathrm{ECA}({\mathrm{DWConv}}_{7\times 7}\left(f_{\mathrm{input}}\right)),f_{\mathrm{DDEM}}\in R^{C_{\mathrm{in}}\times H_{x\_\mathrm{out}}\times W_{x\_\mathrm{out}}}$$

The complete process of the object detection head is as follows:

Firstly, the multi-scale features $P_{x\_\mathrm{out}}\in R^{C_{x\_\mathrm{out}}\times H_{x\_\mathrm{out}}\times W_{x\_\mathrm{out}}},\;x\in \left\{3,4,5\right\}$, which are fed into the classification branch and the regression branch, are first unified in the channel dimension by 1×1 convolution, as shown in Equation (23). The features $f_{\mathrm{stem}}$ after channel alignment are sent to DDEM for feature enhancement to obtain $f_{\mathrm{cls}\_\mathrm{feat}}$ and $f_{\mathrm{reg}\_\mathrm{feat}}$. The two branches are independent and do not share weights, enabling them to focus more on their respective tasks and improving the overall performance.

(23) $$f_{\mathrm{stem}}={\mathrm{Conv}}_{1\times 1}\left(P_{x\_\mathrm{out}}\right),f_{\mathrm{stem}}\in R^{C_{\mathrm{in}}\times H_{x\_\mathrm{out}}\times W_{x\_\mathrm{out}}}$$

Then, the prediction results are output through 1×1 convolution, and the formulas are shown in Equations (24)–(26):

(24) $$\begin{array}{c} f_{\mathrm{cls}\_\mathrm{out}}={\mathrm{Conv}}_{1\times 1}\left(f_{\mathrm{cls}\_\mathrm{feat}}\right),f_{\mathrm{cls}\_\mathrm{out}}\in R^{N_{\mathrm{cls}}\times H_{x\_\mathrm{out}}\times W_{x\_\mathrm{out}}} \end{array}$$

(25) $$\begin{array}{c} f_{\mathrm{reg}\_\mathrm{out}}={\mathrm{Conv}}_{1\times 1}\left(f_{\mathrm{reg}\_\mathrm{feat}}\right),f_{\mathrm{reg}\_\mathrm{out}}\in R^{4\times H_{x\_\mathrm{out}}\times W_{x\_\mathrm{out}}} \end{array}$$

(26) $$\begin{array}{c} f_{\mathrm{obj}\_\mathrm{out}}={\mathrm{Conv}}_{1\times 1}\left(f_{\mathrm{reg}\_\mathrm{feat}}\right),f_{\mathrm{obj}\_\mathrm{out}}\in R^{1\times H_{x\_\mathrm{out}}\times W_{x\_\mathrm{out}}} \end{array}$$

Finally, the bounding box feature $f_{\mathrm{reg}\_\mathrm{out}}$, the target confidence feature $f_{\mathrm{obj}\_\mathrm{out}}$, and the category feature $f_{\mathrm{cls}\_\mathrm{out}}$ are concatenated and fused along the channel dimension to obtain the prediction result as shown in Equation (27):

(27) $$f_{\det\_\mathrm{out}}=\mathrm{Concat}\left(f_{\mathrm{cls}\_\mathrm{out}},f_{\mathrm{reg}\_\mathrm{out}},f_{\mathrm{obj}\_\mathrm{out}}\right),f_{\det\_\mathrm{out}}\in R^{\left(5+N_{\mathrm{cls}}\right)\times H_{x\_\mathrm{out}}\times W_{x\_\mathrm{out}}}$$

### 3.6. Segmentation Head

The segmentation head outputs a total of 9 categories, namely multi-category water-surface targets, drivable area, and background. In this paper, the five-layer multi-scale features $\left\{P_{3\_\mathrm{out}},P_{4\_\mathrm{out}},P_{5\_\mathrm{out}},P_{6\_\mathrm{out}},P_{7\_\mathrm{out}}\right\}$ output by the BiFPN neck network and the shallow features $P_{2\_\mathrm{out}}$ obtained by fusing the backbone network are used as the input of the segmentation head.

Considering that $P_{2\_\mathrm{out}}$ possesses rich fine-grained semantic information and low-level detailed features can improve the accuracy of segmentation prediction, all features are uniformly scaled to the resolution of $P_{2\_\mathrm{out}}$ level, and the low-level $P_{2\_\mathrm{out}}$ features output by the backbone network are involved in the final feature fusion. The overall process is shown in Figure 1.

Firstly, perform upsampling operations on each layer of features to uniformly output feature maps with the size of $\left(64,\frac{W}{4},\frac{H}{4}\right)$ as $P_{2\_\mathrm{out}}$. Secondly, also send the shallow features $P_{2\_\mathrm{out}}$ obtained from the fusion of the backbone network into the convolution layer. Then, add all the hierarchical features element-wise for fusion to obtain a more optimal comprehensive feature. Finally, restore the feature maps to the original image size (3,W,H) and output the predicted probabilities for each pixel as belonging to different categories.

### 3.7. Multi-Task Training Strategy

The task training strategy includes two parts: the basic loss function and the multi-task optimization balancing strategy.

#### 3.7.1. Basic Loss Function

The basic loss function needs to consider two types of losses: the object detection loss and the semantic segmentation loss for the joint of the object and the drivable area, in order to simultaneously achieve the two perception tasks of object detection and the joint semantic segmentation of the object and the drivable area. The object detection loss $L_{\det}$ is as shown in Equation (28).

(28) $$L_{\det}=\alpha_{1}L_{\mathrm{bbox}}+\alpha_{2}L_{\mathrm{obj}}+\alpha_{3}L_{\mathrm{cls}}$$

In Equation (28), $L_{\mathrm{bbox}}$ represents the GIoU loss [31], which takes into account the overlap ratio, distance, and aspect ratio between the predicted box and the ground-truth bounding box; $L_{\mathrm{obj}}$ and $L_{\mathrm{cls}}$ use Focal loss [32] to make the network focus on mining difficult samples; $\alpha_{1}$, $\alpha_{2}$, and $\alpha_{3}$ are manually tuned weighting parameters.

The total loss function $L_{\mathrm{seg}}$ for semantic segmentation is shown in Equation (29).

(29) $$L_{\mathrm{seg}}=L_{\mathrm{focal}}+L_{\mathrm{dice}}$$

In Equation (29), $L_{\mathrm{focal}}$ represents the Focal Loss which is used to reduce the loss weight for simple background pixels, focusing on difficult samples such as distant small obstacles and alleviating the imbalance in the number of foreground and background pixels in water-surface scenes; $L_{\mathrm{dice}}$ is the Dice Loss [33] which calculates the overlap between the predicted mask and the ground-truth mask to optimize the segmentation contour and solve the problem of insufficient gradients caused by too few foreground pixels.

#### 3.7.2. Multi-Task Optimization Balance Strategy

The loss magnitudes vary greatly for different tasks during the training process. If a simple summation of losses is adopted for optimization, the network parameters will be updated in a way that favors the tasks with larger loss values, resulting in an imbalance in the training of different tasks. Therefore, a multi-task training strategy with homoscedastic uncertainty [34] is employed. The total loss formula for panoptic perception multi-tasks is as shown in Equation (30):

(30) $$L_{\mathrm{total}}=\left(e^{-\lambda_{1}}L_{\det}+\lambda_{1}\right)+\left(e^{-\lambda_{2}}L_{\mathrm{seg}}+\lambda_{2}\right)=\sum_{i=1}^{T}\left(e^{-\lambda_{i}}L_{i}+\lambda_{i}\right)$$

In Equation (30), T=2 represents the two perception tasks of detection and segmentation. $\lambda_{1}$ and $\lambda_{2}$ are independent learnable log-variance parameters for the two tasks, where $\lambda_{i}=\log\sigma_{i}^{2}$. The exponential term $e^{-\lambda_{i}}$ adaptively adjusts the contribution weight of each task loss during training, while the $\lambda_{i}$ term acts as a regularizer to prevent the task weight from vanishing. This weighting mechanism removes the requirement of manually tuning loss weights.

This dynamic weighted equalization strategy has two significant advantages: firstly, it eliminates the need for repeated experiments and adjustments of task weights for various water-surface data sets, thereby reducing the cost of parameter tuning and enhancing the model’s cross-scenario generalization ability; secondly, it effectively suppresses the problem of single-task dominance in gradient updates, avoiding excessive detection loss that causes blurred segmentation contours and excessive segmentation loss ratio that leads to missed detections, and achieving synchronous and stable convergence of the two tasks of object detection and pixel segmentation.

## 4. Experiments

### 4.1. Data Set

Two radar-visual fusion-based water-surface perception datasets, namely WaterScenes [35] and Flow-RI [36], were selected to verify the performance of RVFNet. The nuScenes v1.0-trainval autonomous driving dataset [37] was chosen to validate the generalization ability of the model. In terms of data division, a uniform frame-level random partitioning strategy is adopted for the three datasets: all frames of each dataset are randomly shuffled, and then divided into the training set, validation set and test set in a ratio of 7:1:2. The descriptions of the datasets are as follows:

WaterScenes Dataset: This dataset covers various time conditions (daytime, nightfall, night), lighting conditions (normal, dim, strong), weather conditions (sunny, overcast, rainy, snowy), and water-surface conditions (river, lake, canal, moat), providing data from multiple sensors. It includes a 4D millimeter-wave radar, a monocular camera, GPS and a set of 10-axis inertial navigation AHRS (IMU)—consisting of a three-axis gyroscope, a three-axis accelerometer, a three-axis magnet and a barometer—to track the attitude and motion information of the USV. It is currently the largest benchmark dataset for multi-task perception of water-surface that integrates vision and 4D mmWave radar. The dataset contains 54,120 frames of images and 202,807 targets; for the tasks of object detection and target semantic segmentation, seven types of targets are set, namely pier, buoy, sailor, ship, boat, vessel, and kayak. The dataset can perform pixel-level annotation for navigable water areas and supports the task of navigable water area segmentation.

Flow-RI Dataset: This dataset is a radar-visual fusion dataset specifically designed for the detection of small floating debris on water-surface. It only includes the detection of one type of object, namely bottle. There are a total of 2227 images, with 5230 target instances.

nuScenes v1.0-trainval Autonomous Driving Dataset: The rapid development of autonomous driving technology is supported by high-quality datasets. As one of the most comprehensive multimodal datasets for autonomous driving, nuScenes was published by the nuScenes team in Boston and Singapore. nuScenes v1.0-trainval is a training and validation version of the complete nuScenes dataset. It is collected by six cameras, one lidar, five millimeter-wave radars, GPS and IMU simultaneously, covering various complex traffic environments such as night and rain. The complete nuScenes dataset contains about 1.4 million camera images, 1.4 million radar scans, and more than 1.4 million 3D object frames. The object detection task is divided into 10 categories: cars, trucks, construction vehicles, buses, trailers, barriers, motorcycles, bicycles, pedestrians, and traffic cones. According to the official setting, the dataset is divided into 850 scenes for training and validation, and 150 scenes for testing.

Considering that the size of the full version of the original data is more than 300 GB, directly using all the data will bring a significant burden in terms of storage and read efficiency, and the cross-scene experiment positioning of this paper is to test the generalization trend of the model in the road scene, rather than pursuing the absolute accuracy on this dataset. Therefore, the nuScenes v1.0-trainval01_blobs volume is selected to build the cross-scenario evaluation set in this paper. The volume contains 20,256 images captured by six cameras, and 125,558 labeled object instances, covering urban roads, day and night changes, and various weather conditions. The data distribution of nuScenes is consistent with the overall data set, which is fully representative. Another reason for choosing this volume is that it can complete the training and evaluation of all comparison methods under limited computing and storage resources, so that it is easy to compare methods under a uniform data partition and input resolution, so as to ensure the comparability of results. At the same time, this experiment can provide a guiding conclusion for the subsequent large-scale training and domain adaptation validation on the complete nuScenes. In summary, this paper constructs a cross-scene evaluation set based on the multi-camera image data in nuScenes v1.0-trainval01_blobs to test the generalization performance of the model in road scenes.

### 4.2. Settings for Training and Evaluation

Training settings for the WaterScenes dataset: From the zero-training stage, the sizes of the visible light images and the radar pseudo-images were uniformly scaled to 384 × 384. The batch size was set to 32. All models were trained for 100 rounds. The initial learning rate was set to 0.03. Stochastic Gradient Descent (SGD) with a momentum coefficient of 0.937 was used as the optimizer and the cosine annealing learning rate scheduler. During the training process, Exponential Moving Average (EMA) was used to alleviate the overfitting phenomenon of the model, effectively improving the generalization ability and stability of the model. All training and testing tasks were completed using an RTX 5090 GPU (NVIDIA Corporation, Santa Clara, CA, USA).

Training settings for the Flow-RI dataset and the nuScenes v1.0-trainval dataset: All the training parameters, data preprocessing, and hardware environment settings for these two datasets are exactly the same as those in the WaterScenes dataset training scheme. During the training process, only the object detection branch is optimized.

Evaluation metrics for the WaterScenes dataset: For the object detection task, two metrics, ${\mathrm{mAP}}_{50}$ and mAP_50–95_, are used for comprehensive assessment. AP (Average Precision) refers to the area enclosed by the precision--recall curve under the Intersection over Union (IoU) threshold. ${\mathrm{AP}}_{50}$ represents the AP calculated when the IoU threshold is 0.50. The average of ${\mathrm{AP}}_{50}$ for all categories is obtained to calculate ${\mathrm{mAP}}_{50}$; the average of AP for all categories is calculated to obtain mAP_50–95_. Here, AP is calculated at each 0.05 step from IoU threshold 0.5 to 0.95. For the target semantic segmentation and navigable water area segmentation tasks, mIoU is uniformly used as the core evaluation metric for segmentation performance.

Since only the object detection task was conducted for validation on the Flow-RI and nuScenes v1.0-mini datasets, ${\mathrm{mAP}}_{50}$ and mAP_50–95_ were adopted as the evaluation indicators for detection accuracy.

### 4.3. The Overall Performance of Various Advanced Models on Different Tasks

Table 1 presents the experimental results of RVFNet and 15 advanced models on the WaterScenes test set, verifying the comprehensive performance of RVFNet in water-surface perception scenarios. In order to ensure a fair comparison of the experiment, this study selects the classical model with similar parameters and calculation amount and the current best baseline model to participate in the experiment. To ensure the fairness of the comparison, all the participating comparison models unify the optimizer, learning rate scheduling strategy, training rounds, batch size, input resolution and data augmentation strategy. Because the task head structure of the single-task model and the multi-task model are naturally different, the loss function cannot be completely consistent. For each baseline model, this paper uses the loss function design recommended by the original paper, and the rest of the training hyperparameters remain the same. All the models participating in the evaluation are divided into two categories: single-task models and multi-task models. Among the single-task semantic segmentation models, there are Segformer-B0 [38], PSPNet [39], YOLOv26-N-sem [40], DeepLabV3+ [41], and the most advanced radar-visual fusion semantic segmentation model RSOS-Net [42]; the single-task object detection models include YOLOv8-N [43], YOLOv9-T [44], YOLOv10-N [45], YOLOv11-N [46], YOLOv12-N [47], and YOLOv26-N [40]; in the multi-task models, classic networks for visual panoptic perception, such as YOLOP [48] and HybridNets [49], as well as radar-visual fusion networks, such as Achelous (MV-GDF-PN-S2) and ASY-VRNet, were selected. Through various comparisons, the advantages of RVFNet in water-surface panoptic perception were fully verified.

**Figure 5:**
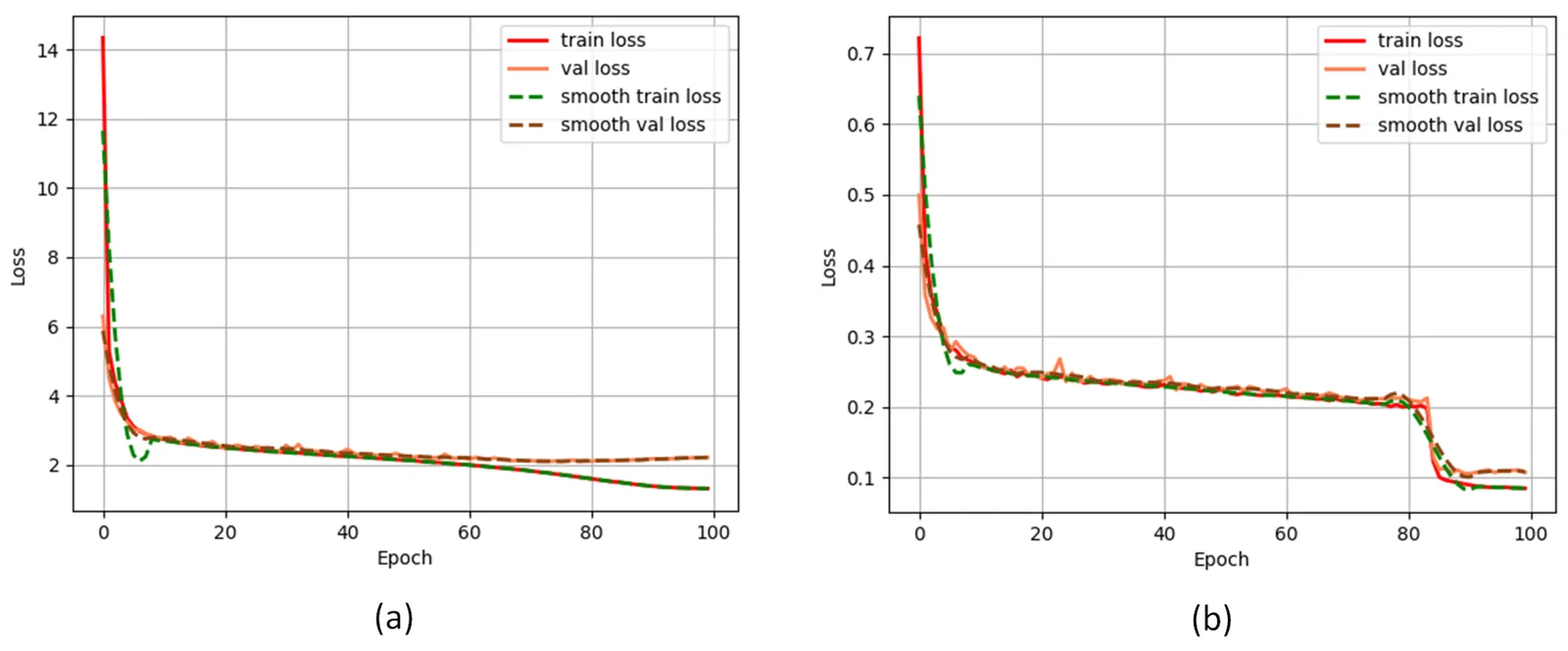
Training and validation loss of RVFNet. (**a**) Object detection. (**b**) Segmentation of targets and drivable area.

As can be seen from Figure 5, RVFNet is able to converge normally during the multi-task training process. As can be seen from Table 1, among all the comparison models, the parameter quantity of RVFNet is not the lowest, but its GFLOPs reach the lowest level. This indicates that RVFNet maintains the multi-task perception performance while achieving higher computational efficiency. It is suitable for deployment on embedded devices with limited computing power and can support real-time inference by edge devices of USVs.

Table 1 shows that the single-task model YOLOv8-N achieves the best detection performance, with mAP_50–95_ of 46.6% and ${\mathrm{mAP}}_{50}$ of 72.0%; the multi-task model Achelous (MV-GDF-PN-S2) achieves the second-best detection performance, with mAP_50–95_ of 46.5% and ${\mathrm{mAP}}_{50}$ of 77.9%. RVFNet achieves the following two indicators: 46.7% and 79.0%. mAP_50–95_ of RVFNet is 0.1% higher than the best single-task model YOLOv8-N and 0.2% higher than the multi-task model Achelous (MV-GDF-PN-S2); meanwhile, ${\mathrm{mAP}}_{50}$ of RVFNet is 1.1% higher than Achelous (MV-GDF-PN-S2) and 7.0% higher than YOLOv8-N.

From Table 1, it can also be seen that the task of drivable area segmentation is relatively easy. ${\mathrm{mIoU}}_{d}$ indicators of all comparison models are concentrated in the range of 98.5% to 99.0%, with very little performance difference. Among them, RVFNet achieved a relatively superior accuracy of 98.8%. The task of water-surface object segmentation requires higher feature extraction capabilities. The performance differences among the models are significant, but ${\mathrm{mIoU}}_{t}$ of RVFNet reached 84.0%, which is better than all comparison models. Compared with the mainstream radar-visual fusion model ASY-VRNet, its performance has improved by 5%.

In summary, RVFNet achieves the best performance for both object detection and object semantic segmentation tasks while maintaining a low computational cost. It effectively balances the model’s computational overhead with multi-task perception accuracy, making it more suitable for the embedded deployment of USVs and the real-time perception requirements in complex water environment scenarios.

### 4.4. RVFNet Ablation Experiments

In this paper, multiple sets of ablation experiments are carried out for RFEM and MFAFM, and the influence of removing radar features in RVFNet on the network is verified. To ensure reliable conclusions, each configuration was repeated three times independently under the same training settings, and the mean and standard deviation of each index are reported in Table 2.

As can be seen in Table 2, MFAFM resulted in the most significant accuracy improvement: after replacing MFAFM with a simple element-wise addition, it resulted in a 0.7% reduction in mAP_50–95_ and 0.3% reduction in ${\mathrm{mIoU}}_{t}$, the largest reductions of all ablation configurations, with standard deviations of only 0.0% to 0.1% for three repeated experiments. MFAFM can learn to generate dynamic fusion weights that are strongly related to the spatial position. The contribution of dual-modal features is adjusted according to the changes of the water scene. The weight proportion of the radar is enhanced in the image degradation area, and the feature response corresponding to the invalid echo is suppressed in the radar clutter interference area, so as to achieve the dynamic complementarity between the image and the radar modality. However, the element-by-element addition with fixed weight cannot achieve this dynamic adjustment, but makes the radar noise unfiltered to pollute the visual features, resulting in greater accuracy loss.

When RFEM was deleted, mAP_50–95_ decreased by 0.3%, ${\mathrm{mIoU}}_{t}$ decreased by 0.3%, and ${\mathrm{mAP}}_{50}$ decreased by 0.1%, indicating that RFEM promotes the interaction and complementary coupling of multi-dimensional physical features such as distance and velocity, enriches feature space discrimination, and achieves clutter suppression in channel dimension. The effective representation ability of radar point cloud is strengthened. If RFENet and MFAFM are further removed, the model deforms into a pure visual single-modal network, with a decrease of 0.6% in ${\mathrm{mAP}}_{50}$, 0.6% in ${\mathrm{mIoU}}_{t}$ synchronization, and 0.3% in mAP_50–95_, which directly reflects the complementary gain of radar modality to visual features. Notably, the decrease in mAP_50–95_ is comparable to that when RFEM is removed alone, indicating that the dynamic weight of MFAFM can suppress noise in the unrefined radar features to some extent when MFAFM is retained. However, the range and velocity information provided by the radar branch is still irreplaceable, and the gain is more direct in ${\mathrm{mAP}}_{50}$ and ${\mathrm{mIoU}}_{t}$.

In addition, ${\mathrm{mIoU}}_{d}$ is stable at 98.8% under each configuration, indicating that this index has become saturated and is not sensitive to the change of network structure. Therefore, the contribution of each module is mainly reflected in mAP and ${\mathrm{mIoU}}_{t}$. Synthesizing all ablation results, it can be seen that deleting RFEM, replacing MFAFM, and cancellingthe supplement of radar features will cause the synchronous attenuation of the accuracy of the two tasks of target detection and target semantic segmentation. And the standard deviation of all indicators does not exceed 0.2%, indicating that the above conclusions are stable in repeated experiments. The synergy between RFEM and MFAFM ensures the high-precision output of water surface perception.

### 4.5. RFEM Ablation Experiments

To gain a deeper understanding of the individual contributions of each component within RFEM, this section conducts fine-grained ablation experiments on the four key components of RFEM based on the complete RVFNet. Channel Shuffle, ECA, residual connection, and Deformable Convolution are removed one by one, with only one component being changed at a time while keeping the rest of the structure and training settings unchanged. The results are shown in Table 3.

The experimental results show that each of the four components contributes differently to the performance. After removing Channel Shuffle, mAP_50–95_ decreased from 46.7% to 46.2%, ${\mathrm{mAP}}_{50}$ dropped to 78.4%, and ${\mathrm{mIoU}}_{t}$ decreased to 83.6%. The detection indicators experienced the largest drop among all four configurations. The reason is that the fused radar features are still isolated along the three physical channels of distance, speed, and reflection intensity, lacking the blocked information flow across dimensions after channel reorganization, and the interaction coupling of multiple physical features is weakened, directly affecting the expression of detection features. After removing ECA, mAP_50–95_ dropped to 46.0%, the lowest among all four configurations, indicating that the lightweight channel attention has a clear filtering effect on the ineffective channels corresponding to radar clutter; without this mechanism, the feature signal-to-noise ratio decreased, and the response of the target-related channel was diluted by the clutter response. After removing the residual connection, mAP_50–95_ dropped to 46.5%, but the decrease in ${\mathrm{mAP}}_{50}$ was the most obvious, indicating that the loss of shallow radar details mainly affects the detection confidence and location recall of the target, and the residual structure’s role in maintaining details and gradient stability has been verified. After removing Deformable Convolution, the dual branches degenerated into two identical standard convolution branches, mAP_50–95_ dropped to 46.4% and ${\mathrm{mAP}}_{50}$ dropped to 78.3%, and the network lost its adaptive modeling ability for the sparse and irregular spatial morphology of radar point clouds, verifying the necessity of Deformable Convolution in radar feature enhancement.

Overall, the absence of any single component would lead to a consistent decline in detection and segmentation performance. The reduction in each component is concentrated between 0.3% and 0.7% for mAP_50–95_ and between 0.1% and 0.4% for ${\mathrm{mIoU}}_{t}$. This indicates that the gains of RFEM are not attributed to a single component but are jointly formed by the collaborative effects of spatial form adaptation, cross-channel information interaction, channel selection, and detail preservation in four dimensions. This result is in line with the conclusion in Section 4.4 Table 2, where removing the entire RFEM resulted in a 0.3% decrease in mAP_50–95_ and a 0.3% decrease in ${\mathrm{mIoU}}_{t}$: the overall ablation defines the total contribution of RFEM, and this section’s fine-grained ablation further decomposes the total contribution into each component, indicating that the design of RFEM does not have redundant components.

### 4.6. Missing Modality Ablations

During actual navigation, the cameras and radar sensors carried by the unmanned vessel may experience performance degradation or even complete failure due to lens contamination, hardware malfunctions, or interference from harsh environments. To verify the usability and robustness of RVFNet under the condition of modal absence, this section conducts an ablation experiment on modal absence. Following the modal random zeroing strategy in MAMMOTH [50], the experiment adopted a two-stage setup where the training stage is randomly set to zero and the inference stage is completely set to zero, to simulate the situation where a certain sensor fails or becomes faulty during use, and to test the perception performance of the model when relying solely on a single mode for inference. The results are shown in Table 4.

The experimental results are highly consistent with the modal characteristics. When the RGB mode is set to zero, the model completely loses pixel-level texture, color, and contour and other semantic information. The target detection indicators mAP_50–95_ and ${\mathrm{mAP}}_{50}$ both drop to 0, meaning that the model is unable to complete the target detection task; although the semantic segmentation task still has some outputs, ${\mathrm{mIoU}}_{t}$ drops sharply to 12.1% and ${\mathrm{mIoU}}_{d}$ drops to 40.3%, and the prediction of the drivable area almost fails. This result indicates that the visual mode carries the main semantic information required by RVFNet, and the sparse geometric information of the radar alone cannot support detection and fine segmentation.

When the radar mode is set to zero, the model degenerates into pure visual perception. The mAP_50–95_ drops from 46.7% to 23.8%, ${\mathrm{mAP}}_{50}$ decreases to 62.1%, and ${\mathrm{mIoU}}_{t}$ drops to 77.8%. However, the model has not completely failed. The detection and segmentation performance still remains at a usable level. This is consistent with the ablation conclusion in Section 4.4 Table 2: In Table 2, removing all radar features also causes a 0.3% decrease in mAP_50–95_ and a 0.3% decrease in ${\mathrm{mIoU}}_{t}$, indicating that the addition of radar features has a consistent positive contribution to both detection and segmentation; the difference is that the modal absence experiment in Table 4 further simulates the extreme scenario of sensor failure, and its performance decline is more significant, intuitively quantifying the compensatory value of the radar mode under visual degradation conditions.

By comparing the two missing cases, it can be found that the tolerance of RVFNet to radar loss is much higher than that to visual loss. This asymmetry results from the information division of the two modalities: image provides fine-grained semantics for target recognition and segmentation, and radar provides stable physical cues such as distance and speed under harsh conditions. When the vision is available, the accuracy of the model is the highest. When the vision is degraded, the radar weight is automatically improved under the dynamic adjustment of MFAFM, and the performance of the model is mildly reduced. When the radar fails, the model can still maintain the basic perception ability based on vision.

### 4.7. Comparison Experiment of Visual and Radar Encoders

To verify the feature extraction advantage of RVFNet, under the condition where the number of channels remains completely consistent, we replaced it with the RCNet (Radar Map Encoder) of Achelous (MV-GDF-PN-S2) and the EfficientNet-B0 backbone for comparative experiments. The experimental results are shown in Table 5.

The experimental data show that when RFENet is replaced by RCNet, ${\mathrm{mAP}}_{50-95}$ of object detection decreases by 1.0%, ${\mathrm{mAP}}_{50}$ decreases by 0.8%, and ${\mathrm{mIoU}}_{t}$ of water-surface object segmentation slightly decreases by 0.1%. Although RCNet introduces deformable convolution to adapt to the spatial sparsity of radar, its coding process is still limited to the degree of correlation between multiple physical attributes such as radar range and velocity, and lacks a special suppression mechanism for radar clutter. RFENet relies on the RFEM module to realize multi-physics information flow and clutter filtering, so the extracted radar features have better effects; If it is replaced by the general lightweight backbone EfficientNet-B0, the model performance undergoes a significant decline. mAP_50–95_ drops by 19.7%, ${\mathrm{mAP}}_{50}$ decreases by 34.2%, the object segmentation ${\mathrm{mIoU}}_{t}$ decreases by 22.6%, and only the indicators for the task of identifying navigable water areas show a slight decline. It shows that the general CNN backbone is designed for RGB images, and the traditional convolutional sampling mode does not match the sparse and irregular distribution characteristics of radar pseudo-images, and cannot effectively use the radar physical attributes, so it cannot be directly reused as a radar encoder.

To verify the feature extraction advantages of the visual feature extraction network of RVFNet, under the condition where the number of channels remains completely consistent, the main branches were replaced with EdgeNext [51], EdgeViTs [52], GhostNetV2 [53], MobileNetV3 [54], MobileViT [55], and ShuffleNetV2 [56] respectively for comparative experiments. The experimental results are shown in Table 6.

Table 6 shows the experimental data indicating that all the lightweight visual backbone replacement methods have led to a decline in the overall performance of the model. Among them, EdgeNext caused the most significant performance degradation. RVFNet outperformed the best-performing MobileVit in the comparison group by 1.2% in mAP_50–95_, 1.7% in ${\mathrm{mAP}}_{50}$, and 0.9% in ${\mathrm{mIoU}}_{t}$, suggesting that the EfficientNet-B0 visual backbone selected in this paper is the most effective in extracting visual features for water-surface scenes.

### 4.8. Comparison Experiment of Fusion Methods

This paper conducted a comparative experiment between MFAFM and several mainstream cross-modal fusion algorithms, including element-wise addition fusion (Addition), the fast fusion module in Achelous, Multi-Head Cross Attention (MHCA) [57], and TokenFusion [58]. All experiments uniformly fixed the network backbone, neck, and detection head, only replacing the fusion module. The experimental results are shown in Table 7.

The results in Table 7 show that the MHCA fusion module relying on the global receptive field has the worst overall performance, and the multi-head cross-attention will establish cross-modal dependence on all positions of the feature map. There are a lot of clutter and invalid blank points in the radar pseudo-image, and the global mechanism is easy to introduce the wrong association between noise features, and it will weaken the feature response of small-size water-surface targets. The performance of detection and segmentation is degraded. The general fusion module TokenFusion is only the same as MFAFM in some indicators, and there is an obvious gap in the comprehensive accuracy. TokenFusion focuses on the semantic interaction at the Token level, lacks the fine-grained judgment of modal contribution at the pixel level, and is difficult to adapt to the spatial non-uniform interference characteristics of the water-surface scene. MFAFM achieves the best performance on the two core tasks of object detection and water-surface object segmentation, and the overall performance is better than the other comparison fusion methods. This result confirms the necessity of designing spatial channel-pixel-level joint adaptive fusion mechanism for USV complex scenes. MFAFM achieves effective complementarity between visual texture and radar physical information through dual model dynamic weights, which is more suitable for water-surface perception tasks.

### 4.9. Comparative Experiments on Cross-Domain Generalization

In order to fully verify the generalization ability and scene adaptability of RVFNet, this paper conducts cross-domain comparative experiments in the Flow-RI dataset of the water-surface scenario and the nuScenes v1.0-trainval dataset of the general driving scenarios. The experimental results are shown in Table 8 and Table 9.

#### 4.9.1. Comparison of Flow-RI Water-Surface Floating Small Waste Data Sets

The results in Table 8 indicate that for the detection scenario of small floating garbage on the water-surface, YOLOv8-N and YOLOv11-N perform relatively well in the pure visual single-modal model, with mAP_50–95_ reaching 40.6% and 40.1% respectively, and ${\mathrm{mAP}}_{50}$ reaching 72.0% and 71.8% respectively; in the radar-visual dual-modal fusion method, Achelous achieves mAP_50–95_ and ${\mathrm{mAP}}_{50}$ of 41.9% and 87.1% respectively, while the performance of ASY-VRNet significantly declines. RVFNet achieves the best results, with mAP_50–95_ and ${\mathrm{mAP}}_{50}$ reaching 42.0% and 87.7% respectively. Both indicators fully surpass all the comparison models, proving that the model in this paper has more accurate and stable target detection capabilities in the water-surface-specific perception scenario.

#### 4.9.2. nuScenes v1.0-trainval01_blobs Autonomous Driving Dataset Comparison

Table 9 shows the cross-domain evaluation results of each model on the nuScenes v1.0-trainval01_blobs test set. Due to the significant domain gap between the road scene and the water scene in the target category, scale distribution and appearance texture, the absolute accuracy of each model generally declines compared with the water dataset. Therefore, this experiment focuses on the relative performance of each method rather than the absolute value.

The overall performance of the pure visual model is weak and unstable: except for YOLOv8-N, which achieves 16.1% mAP_50–95_, the other models are less than 10%, and the relative ranking is inconsistent with the conclusion on the water surface dataset, indicating that it is difficult to maintain a stable target discrimination ability of a single visual modality under cross-domain conditions. In contrast, the dual-modal fusion model has obvious advantages, and the mAP_50–95_ of Achelous reaches 17.8%, which indicates that the geometric and velocity information provided by radar can effectively compensate the degradation of visual features.

RVFNet achieves 40.9% ${\mathrm{mAP}}_{50}$, which is the best among all methods and 1.1% more than Achelous. mAP_50–95_ is 17.5%, which is only 0.3% away from the optimal Achelous, which is at the same level. It should be mentioned that this experiment is not to prove that RVFNet achieves usable accuracy in autonomous driving scenarios, but to examine its relative competitiveness under cross-domain conditions. The results show that the multi-scale feature adaptive fusion mechanism relied on by RVFNet can still play a role under the condition of domain shift, and the complete verification of its generalization ability needs to be targeted training and evaluation on road scene data.

In summary, the experiments in this section show that RVFNet can still maintain competitive performance with similar methods outside the water surface scene, and its fusion mechanism has a certain potential for cross-scene adaptation. It should be pointed out that due to the domain gap and category system difference between the road scene and the water scene, the absolute accuracy of the model on this dataset is still significantly lower than that of the water scene, which indicates that the feature transfer under cross-domain conditions is still worthy of further research. Domain-adaptive training on road data to improve the practical usability of the model in non-water scenarios will be further carried out as a follow-up work.

### 4.10. Comparative Experiments and Visualization Analysis Under Harsh Environments

**Figure 6:**
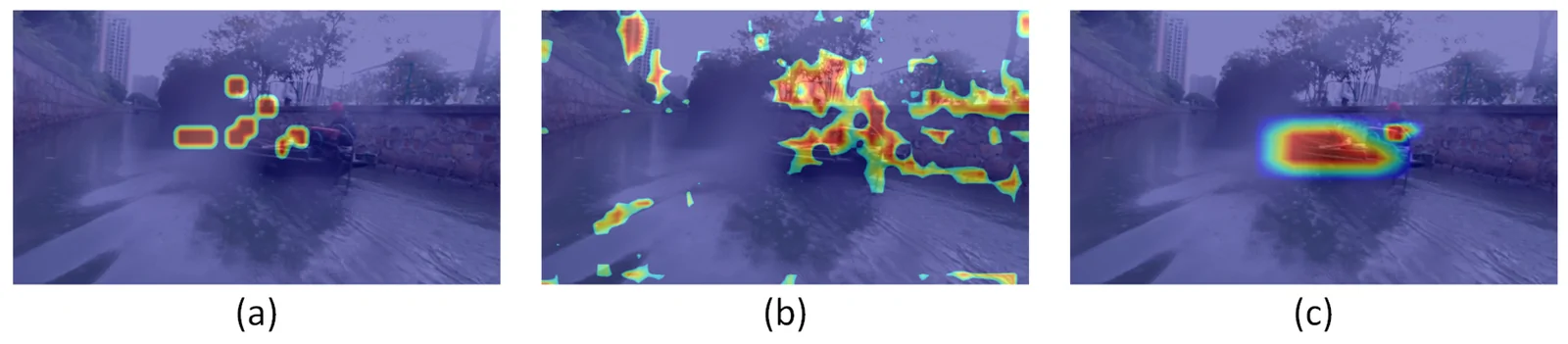
Heatmap visualizations in the thick-fog scenario. (**a**) The radar branch heatmap derived from the third-stage features of the radar backbone; (**b**) the camera branch heatmap derived from the third-stage features of the visual backbone; (**c**) the detection heatmap derived from the final output features of the detection head.

In order to reveal the fusion behavior of MFAFM under the condition of visual degradation, Figure 6 shows the heat map visualization in the dense fog scene, where (a) and (b) are taken from the third stage output features of the radar and visual backbone respectively, reflecting the feature response distribution of each modality; (c) is taken from the final output feature of the detection head, which reflects the discrimination basis of the fusion feature to the target. It can be observed that the response of the camera heatmap (b) is dispersed in the fog mass and areas such as shoreline and trees that are not directly occluded by fog, and there is almost no concern in the position occluded by fog. However, the radar heatmap (a) still maintains an obvious and focused high response in the occluded position, which can perceive the occluded ship target. After the fusion of the two modalities by MFAFM, the detection heatmap (c) forms a concentrated and continuous high response in the occluded target area, successfully locking the target that the camera cannot focus on alone. This phased evolution from single-modal response to fusion detection output intuitively demonstrates the failure of visual modality in dense fog, the robustness of radar modality, and the ability of MFAFM to effectively utilize radar cues, which mutually confirms the quantitative results in Table 10 and Table 11.

**Figure 7:**
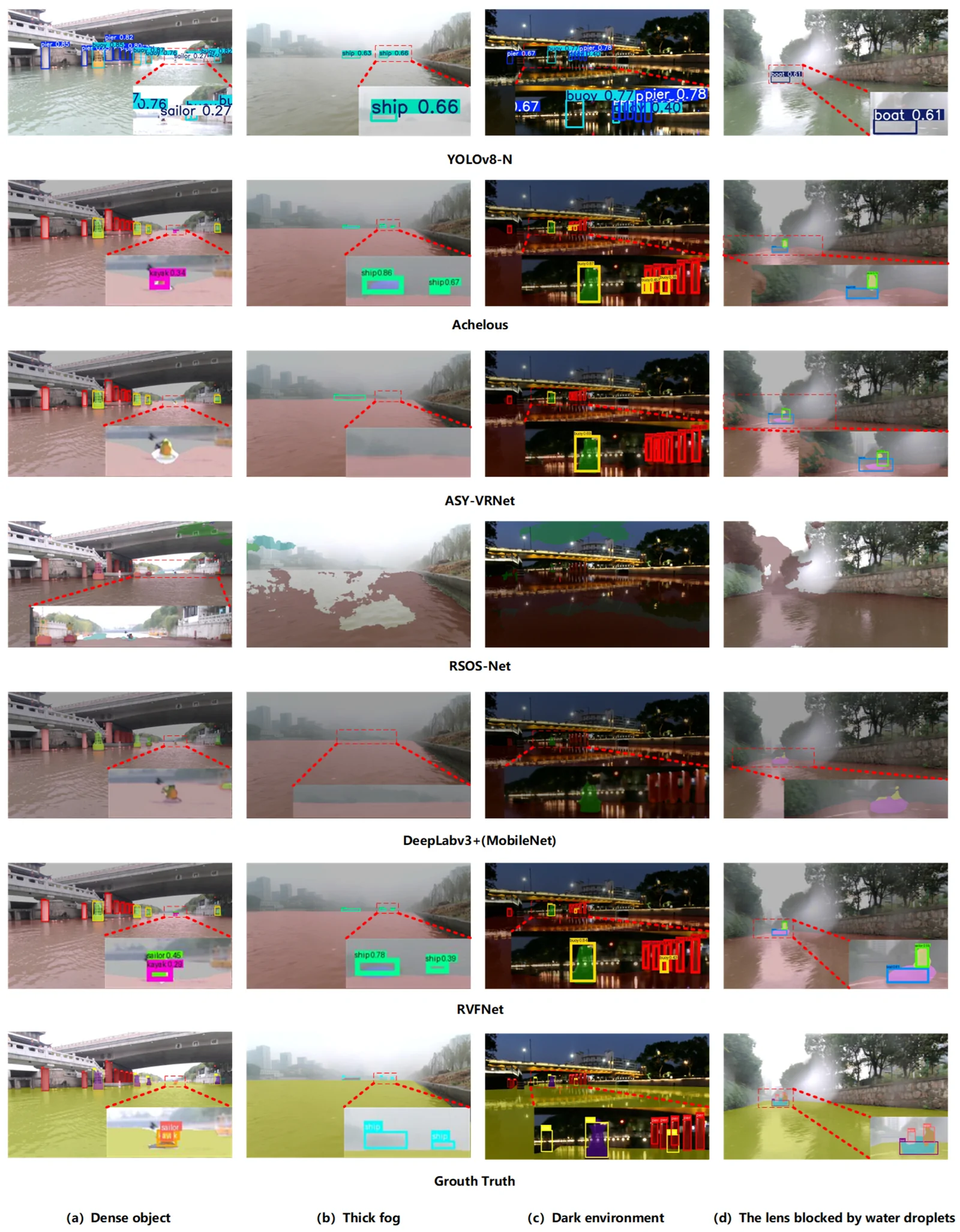
Visualization of RVFNet compared with other models.

To validate RVFNet’s perception capability in complex navigation environments, this section selects four typical challenging scenarios—Adverse Illumination, Adverse Weather, Small Objects, and the Lens Blocked By Water Droplets—for quantitative comparative experiments and qualitative visualization analysis. The results are shown in Table 10 and Table 11, and Figure 7.

Table 10 presents the detection and segmentation performance of each model under Adverse Illumination and Adverse Weather conditions. Under Adverse Illumination conditions, RVFNet achieves mAP_50–95_ and ${\mathrm{mIoU}}_{t}$ of 39.6% and 67.9%, respectively; in contrast, ASY-VRNet’s detection performance drops significantly to 24.7%, indicating its difficulty in effectively utilizing visual features in low-light scenarios. Although Achelous performs slightly better in detection metrics, its segmentation accuracy is only 58.6%, substantially lower than that of RVFNet. Compared with pure segmentation models, RVFNet achieves the best segmentation accuracy while simultaneously performing both detection and segmentation tasks, outperforming RSOS-Net and DeepLabV3+. Under Adverse Weather conditions, RVFNet reaches an mAP_50–95_ of 31.4%, surpassing ASY-VRNet and YOLOv8-N by 13.7% and 1.4%, respectively. Its ${\mathrm{mIoU}}_{t}$ reaches 71.9%, outperforming RSOS-Net and DeepLabV3+. These results demonstrate that incorporating radar modality enables RVFNet to maintain stable detection and segmentation performance under visually degraded conditions such as rain and fog, without mutual suppression between the two tasks.

Table 11 further compares the model performance in two scenarios: small target scenarios and scenarios where the lens is covered by water droplets. In the small target scenarios, the pixel proportions of targets such as water surface buoys and kayaks are low, and the textures are weak, which pose higher requirements for feature extraction capabilities. The ${\mathrm{mAP}}_{50}$ and ${\mathrm{mIoU}}_{t}$ of RVFNet reached 69.4% and 65.0% respectively, both being the optimal values among all the compared models; Achelous had a slightly higher mAP_50–95_ of 32.4%, compared to 29.7% of RVFNet, but its segmentation accuracy was only 55.3%, significantly lagging behind RVFNet and showing an unbalanced overall performance. In the lens covered by water droplets scenarios, the surface texture of the image was directly damaged by the water droplets, and the mAP_50–95_ of RVFNet was 24.2%, which was comparable to 24.6% of the best-performing Achelous, significantly higher than 8.7% of ASY-VRNet and 23.2% of YOLOv8-N; in the segmentation task, its ${\mathrm{mIoU}}_{t}$ reached 49.1%, second only to 51.3% of DeepLabV3+, while ${\mathrm{mIoU}}_{d}$ remained stable at 98.4%. It is worth noting that the degradation of pure visual models in this scenario was significantly greater than that of RVFNet, indicating that when water droplets weaken the visual cues, the compensation effect of radar features on visual degradation becomes prominent, verifying the effectiveness of MFAFM in adaptively increasing the proportion of radar weights in the image degradation area.

Figure 7 presents the visualization results of detection and segmentation for each model in four typical complex scenarios: dense obstacles, thick fog, dark environment, and the lens blocked by water droplets. In the target detection task, the models YOLOv8-N, Achelous, and ASY-VRNet generally have missed detections. Among them, in visual degradation scenarios such as thick fog, dark environment, and the lens blocked by water droplets, RVFNet has the least missed detections and detects more targets. In the dense obstacle scenario, only RVFNet can identify all the targets, while the other comparison models all have missed detections of small targets such as sailors and kayaks.

From the segmentation results of Achelous, ASY-VRNet, RSOS-Net, DeepLabv3+(MobileNet), and RVFNet, it can be seen that the drivable area and target boundary output by RVFNet are smoother and have a higher matching degree, indicating that it has better comprehensive detection and segmentation performance in complex water surface environments.

By comparing the quantitative results in Table 10 and Table 11 with the qualitative results in Figure 7, it can be seen that under challenging conditions such as visual information degradation and complex distribution of small targets, the performance of the pure visual model has significantly declined. However, RVFNet, relying on the enhanced representation of radar multi-physical features by RFEM and the dynamic modal weighting of MFAFM, effectively compensates for the perception ability in the visual failure area. It maintains the optimal or near-optimal comprehensive performance in both target detection and semantic segmentation tasks, verifying the robustness and practical value of the proposed method in harsh navigation environments.

## 5. Conclusions

In response to the shortcomings of radar and visual sensors in feature representation and information fusion, a multi-source information collaborative perception method for USVs in complex environments was studied. A multi-modal multi-task perception framework named RVFNet for water-surface scene was constructed. The research focused on radar information enhancement and multi-modal feature interaction, achieving a transition from single-sensor perception to multi-source information joint perception. RFENet was proposed, which effectively extracted radar spatial attributes and motion-related information, improving the discrimination ability of radar features. MFAFM was designed, enhancing the information complementarity among different sensor features, enabling the model to dynamically adjust different modal information according to the input scene, and effectively improving the stability of the model when facing water-surface reflections, rain and fog weather interference. Experimental results on the WaterScenes, FloW-RI, and nuScenes v1.0-trainval datasets show that RVFNet has superior comprehensive perception performance compared to existing advanced methods, providing a new technical solution for USVs to achieve all-weather autonomous perception. In the future, we will further explore model lightweighting and edge deployment technologies to reduce the number of parameters and computational load, and improve the efficiency of model inference.

## Figures and Tables

**Table 1:** Performances of RVFNet, other multi-task models and single-task models on the WaterScenes test set.

Methods	Sensors	TN	Params (M)	FLOPs (G)	OD		SS	
					mAP_50–95_	mAP_50_	mIoU_t_	mIoU_d_
					(%)	(%)	(%)	(%)
Segformer-B0	C	1	3.7	3.6	-	-	52.6	93.9
PSPNet	C	1	2.4	3.4	-	-	70.4	98.8
YOLOv26-N-sem	C	1	1.6	4.3	-	-	75.5	98.8
DeepLabV3+	C	1	5.2	9.6	-	-	79.2	98.9
RSOS-Net	C + R	1	48.8	31.1	-	-	75.2	**99.0**
YOLOv8-N	C	1	3.0	8.1	46.6	72.0	-	-
YOLOv9-T	C	1	2.0	7.9	36.4	58.2	-	-
YOLOv10-N	C	1	2.7	8.4	38.9	62.4	-	-
YOLOv11-N	C	1	2.6	6.4	46.1	71.8	-	-
YOLOv12-N	C	1	2.6	6.5	36.2	55.8	-	-
YOLOv26-N	C	1	2.4	5.2	38.3	62.4	-	-
YOLOP	C	3	7.9	11.3	37.5	68.3	-	**99.0**
HybridNets	C	3	13.4	5.2	32.7	63.7	-	98.5
Achelous	C + R	5	5.3	8.4	46.5	77.9	70.0	98.8
ASY-VRNet	C + R	5	4.1	4.6	27.6	58.2	79.0	98.5
RVFNet	C + R	3	5.9	3.3	**46.7**	**79.0**	**84.0**	98.8

Note: **TN** stands for task number; **OD** stands for object detection; **SS** stands for semantic segmentation; ${\mathrm{mIoU}}_{t}$ stands for mIoU of targets; ${\mathrm{mIoU}}_{d}$ stands for mIoU of drivable area; C is for camera; R is for radar; “-” stands for this model failed to perform this task, and therefore there are no corresponding evaluation results; Bold values indicate the highest accuracy for each evaluation metric.

**Table 2:** Ablation experiments of RVFNet.

Methods	mAP_50–95_ (%)	mAP_50_ (%)	mIoU_t_ (%)	mIoU_d_ (%)
RVFNet	46.7	79.0	84.0	98.8
-delete RFEM	46.4±0.1	78.9±0.1	83.7±0.2	98.8±0.0
-MFAFM → Addition	46.0±0.0	78.9±0.1	83.7±0.1	98.8±0.0
delete RFENet and MFAFM	46.4±0.2	78.4±0.1	83.4±0.1	98.8±0.0

**Table 3:** Ablation experiments of RFEM.

Methods	mAP_50–95_ (%)	mAP_50_ (%)	mIoU_t_ (%)	mIoU_d_ (%)
RVFNet	46.7	79.0	84.0	98.8
-delete Channel	46.2	78.4	83.6	98.7
-delete ECA	46.0	78.9	83.7	98.8
-delete residual connection	46.5	78.2	83.8	98.7
-delete DConv	46.4	78.3	83.9	98.8

**Table 4:** Available modalities.

Methods	mAP_50–95_ (%)	mAP_50_ (%)	mIoU_t_ (%)	mIoU_d_ (%)
RVFNet	46.7	79.0	84.0	98.8
-delete RGB	0.0	0.0	12.1	40.3
-delete Radar	23.8	62.1	77.8	98.3

**Table 5:** Comparison of radar backbone between RVFNet and other representative backbones.

Methods	mAP_50–95_ (%)	mAP_50_ (%)	mIoU_t_ (%)	mIoU_d_ (%)
Achelous	45.7	78.2	83.9	**98.8**
EfficientNet-B0	27.0	44.8	61.4	98.1
RVFNet	**46.7**	**79.0**	**84.0**	**98.8**

Note: Bold values indicate the highest accuracy for each evaluation metric.

**Table 6:** Comparison of RGB backbone between RVFNet and other representative backbones.

Methods	mAP_50–95_ (%)	mAP_50_ (%)	mIoU_t_ (%)	mIoU_d_ (%)
EdgeNext	31.2	61.2	80.2	98.6
EdgeViTs	42.0	72.8	82.0	98.7
GhostNetV2	43.9	76.0	83.4	98.7
MobileNetV3	43.7	76.2	83.1	98.7
MobileViT	45.5	77.3	83.1	**98.8**
ShuffleNetV2	41.1	73.5	82.5	98.7
RVFNet	**46.7**	**79.0**	**84.0**	**98.8**

Note: Bold values indicate the highest accuracy for each evaluation metric.

**Table 7:** Comparison of fusion methods for vision and radar.

Methods	mAP_50–95_ (%)	mAP_50_ (%)	mIoU_t_ (%)	mIoU_d_ (%)
TokenFusion	45.8	**79.0**	83.9	**98.8**
MHCA	44.9	78.2	83.8	**98.8**
Achelous	45.7	78.6	82.8	98.7
Addition	46.0	78.9	83.7	**98.8**
RVFNet	**46.7**	**79.0**	**84.0**	**98.8**

Note: Bold values indicate the highest accuracy for each evaluation metric.

**Table 8:** Performances of RVFNet, other multi-task models and single-task models on the Flow-RI test set.

Methods	Sensors	mAP_50–95_ (%)	mAP_50_ (%)
YOLOv8-N	C	40.6	72.0
YOLOv9-T	C	9.1	37.2
YOLOv10-N	C	6.5	21.6
YOLOv11-N	C	40.1	71.8
YOLOv12-N	C	4.6	17.9
YOLOv26-N	C	12.0	40.9
Achelous	C + R	41.9	87.1
ASY-VRNet	C + R	15.6	55.8
RVFNet	C + R	**42.0**	**87.7**

Note: Bold values indicate the highest accuracy for each evaluation metric.

**Table 9:** Performances of RVFNet, other multi-task models and single-task models on the nuScenes v1.0-trainval01_blobs test set.

Methods	Sensors	mAP_50–95_ (%)	mAP_50_ (%)
YOLOv8-N	C	16.1	35.5
YOLOv9-T	C	3.5	10.1
YOLOv10-N	C	7.6	18.4
YOLOv11-N	C	7.1	17.3
YOLOv12-N	C	9.2	21.8
YOLOv26-N	C	6.8	18.5
Achelous	C + R	**17.8**	39.8
ASY-VRNet	C + R	6.6	20.4
RVFNet	C + R	17.5	**40.9**

Note: Bold values indicate the highest accuracy for each evaluation metric.

**Table 10:** Comparison of models under Adverse Illumination and Adverse Weather conditions.

	Adverse Illumination				Adverse Weather			
Models	OD		SS		OD		SS	
	mAP_50–95_ (%)	mAP_50_ (%)	mIoU_t_ (%)	mIoU_d_ (%)	mAP_50–95_ (%)	mAP_50_ (%)	mIoU_t_ (%)	mIoU_d_ (%)
RVFNet	39.6	61.7	**67.9**	**98.9**	**31.4**	**60.6**	**71.9**	**98.5**
ASY-VRNet	24.7	51.7	63.8	98.5	17.7	43.7	67.4	97.9
Achelous	**40.1**	**62.2**	58.6	98.7	31.2	60.2	63.2	98.3
YOLOv8-N	39.4	60.1	-	-	30.0	**60.6**	-	-
RSOS-Net	-	-	66.7	98.1	-	-	67.3	97.8
DeepLabV3+		-	67.5	**98.9**	-	-	71.8	98.3

Note: Bold values indicate the highest accuracy for each evaluation metric.

**Table 11:** Comparison of models under Small Object and the Lens Blocked By Water Droplets.

	Small Objects				The Lens Blocked by Water Droplets			
Models	OD		SS		OD		SS	
	mAP_50–95_ (%)	mAP_50_ (%)	mIoU_t_ (%)	mIoU_d_ (%)	mAP_50–95_ (%)	mAP_50_ (%)	mIoU_t_ (%)	mIoU_d_ (%)
RVFNet	29.7	**69.4**	**65.0**	**98.9**	24.2	41.1	49.1	**98.4**
ASY-VRNet	12.6	37.1	54.3	98.5	8.7	28.2	46.0	98.0
Achelous	**32.4**	68.1	55.3	**98.9**	**24.6**	**42.0**	40.6	98.0
YOLOv8-N	28.4	59.2	-	-	23.2	35.2	-	-
RSOS-Net	-	-	40.8	98.1	-	-	49.0	98.2
DeepLabV3+		-	61.6	98.0	-	-	**51.3**	98.1

Note: Bold values indicate the highest accuracy for each evaluation metric.

## Data Availability

Publicly available datasets were analyzed in this study. This data can be found here: WaterScenes dataset: https://github.com/waterscenes/WaterScenes (accessed on 11 October 2025); FloW-RI dataset: https://orca-tech.cn/datasets/FloW/FloW-RI (accessed on 22 June 2026); nuScenes v1.0-mini dataset: https://www.nuscenes.org/nuscenes (accessed on 30 June 2026).

## Abbreviations

The following abbreviations are used in this manuscript:

RFEM	Radar Feature Enhancement Module
RFENet	Radar Feature Extraction Network
MFAFM	Multi-modal Feature Adaptive Fusion Module
USVs	Unmanned Surface Vehicles
ECA	Efficient Channel Attention
AFF	Asymmetric Fair Fusion
IRC	Image-Radar Concatenation
RI	Radar-Image Multiplication
BiFPN	Bidirectional Feature Pyramid Network
FPN	Feature Pyramid Network
GAP	Global Average Pooling
BN	Batch Normalization
DDEM	Depthwise-ECA Detection Enhancement Module
SGD	Stochastic Gradient Descent
EMA	Exponential Moving Average
AP	Average Precision
IoU	Intersection over Union
MHCA	Multi-Head Cross Attention
